# Traumatic injuries in pregnant women: a case of motor vehicle accident for “Ground Round” discussion

**DOI:** 10.5249/jivr.v3i1.28

**Published:** 2011-01

**Authors:** Alireza Ahmadi, Taravat Fakheri, Javad Amini-Saman, Omid Amanollahi, Mahmoudreza Mordi, Maryam Almasi Nasrabadi, Yousef Gholipour, Reza Dehghani, Shahrzad Bazargan-Hejazi

**Affiliations:** ^*a*^Imam Reza Hospital, Kermanshah, University of Medical Sciences, Kermanshah, Iran.; ^*b*^Department of Public Health Sciences, Division of Social Medicine, Karolinska Institute, Stockholm, Sweden.; ^*c*^Department of Psychiatry, College of Medicine, Charles Drew University of Medicine and Science, & David Geffen School of Medicine, UCLA, Los Angeles, CA, USA.

## Abstract

The main objective for introducing this case study is to create a platform from which the importance of road traffic related injuries and traumas can be emphasized and discussed within and across various fields of investigation. The long term goal is to entice public campaign around unmet needs for higher road safety measures to reduce primary, secondary, and tertiary risks of injuries and traumas.

**Case:**

A 28-year-old pregnant woman with a 16-week gestational age fetus was involved in a road car crash resulting in multiple traumas. Evaluation and treatment was initiated in the local Urgent Care Unit and continued in the emergency department and operation room. Patient underwent the following procedures: laparotomy, diverting colostomy, terminating pregnancy, right calcaneal traction and long leg splint, as well as multiple irrigation-debridements. Finally, the wound was left open and the patient was admitted to Intensive Care Unit. We hope that the introduction of this case for a "Ground Round" discussion will stir up a comprehensive discussion regarding the injury and trauma related preventive measures as well as treatment approaches in cases involving pregnant women in car accidents, and will bring about a holistic overview of this issue by the experts in various fields.

## Medical Case in Details

**Figure1: Right side view of crashed car(Peugeot-Paykan):**
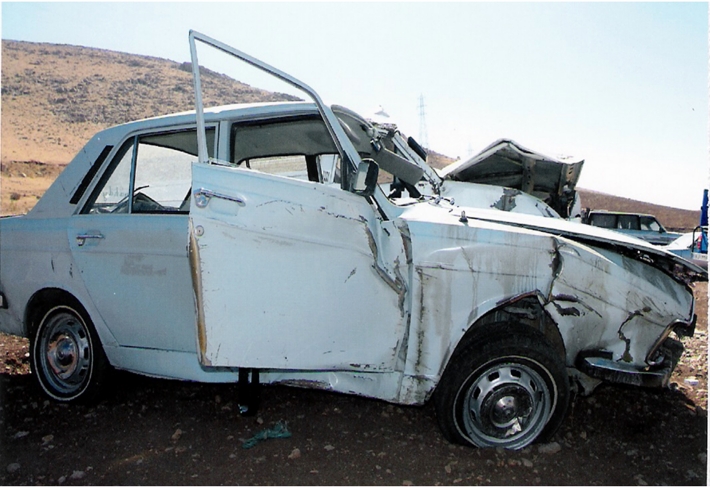


**Figure 2: Front view of crashed car (Peugeot-Paykan):**
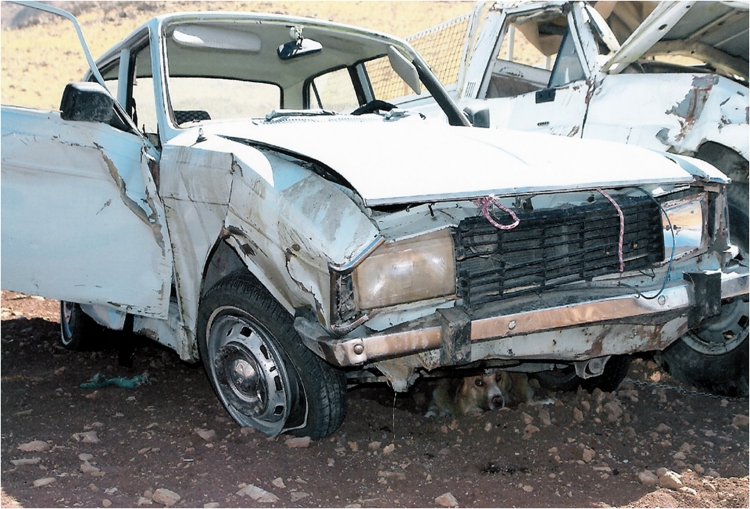


In August 20, 2008, (10:50 AM) a 28-year-old pregnant woman with a 16-week gestational age fetus was involved in a road car crash resulting in multiple traumas. She had two other children; one (a 7 year-old girl) who was diagnosed with Cerebral Palsy and the other (an 18 month–old boy) who was in good health. Gravid=3, term=2, live=2 (G3T2L2). She was seated in the front seat of a midsize sedan car (Peugeot- Paykan) and her seatbelt was fastened(Figure 1,2). Peugeot- Paykan is a common family car in Iran assembled by the Paykan manufacturing with the same body panels as Paykan, however, manufacturing of Peugeot 504 engines has been suspend since 1991. Her brother was the driver. Nobody else was in the car. The cause of accident was speeding causing lost  of  control  and  subsequently  colliding with front long-vehicle. After the accident, she was transferred to a local Urgent Care Unit and received first aid of the ABCDE care included  : airway, breathing, circulation, disability, and exposure. She was then transferred to a 515-bed General Hospital (Imam Reza Hospital) (arrived at 12.30 AM).  The primary examinations revealed patient was pale, no respiratory distress, Glasgow Coma Scale=15, Blood Pressure=90/50 mmHg, Pulse Rate=100/min, and Respirat-ory Rate=20/min. She had two open IV lines and lower extremities were fixed with removable splints.

Moreover, the inferior of the abdomen and perineum areas were lacerated extensively, including vaginal (labia major and minor were involved, and the clitoris was traumatically removed), rectal areas (external sphincter was torn but the mucosal layer of rectum was intact) (Figure 6). The patient's wounds activated bleeding and four extremities pulse could be palpated. Uterine size was about 18 weeks of pregnancy and a transverse supra pubic scar due to previous cesarean section could be seen. Blood sampling was done, and fluid and blood resuscitation were continued.

**Figure 3: Superior and inferior right ramous of pelvic Fracture:**
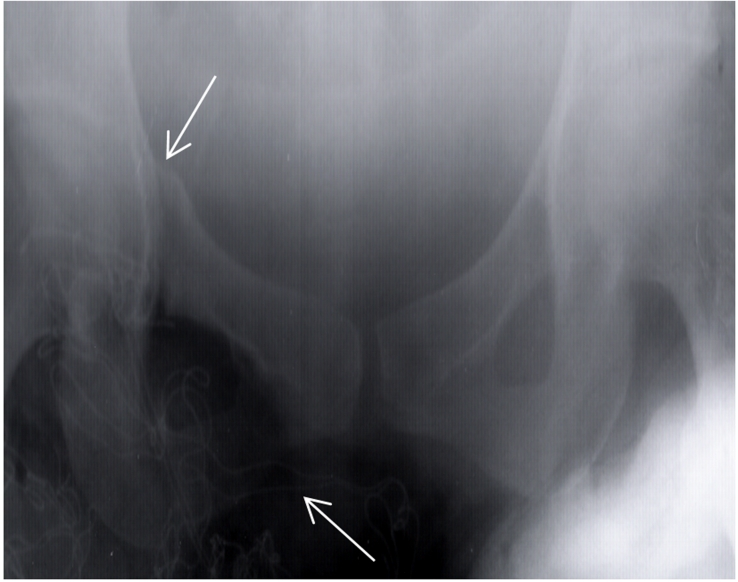


**Figure 4: Proximal of right tibia fracture:**
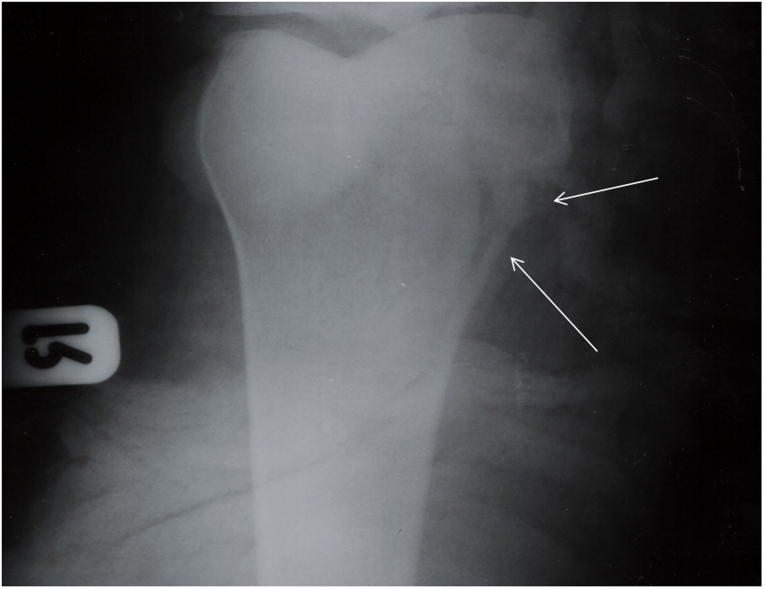


**Figure 5: Shaft of left fibula fracture:**
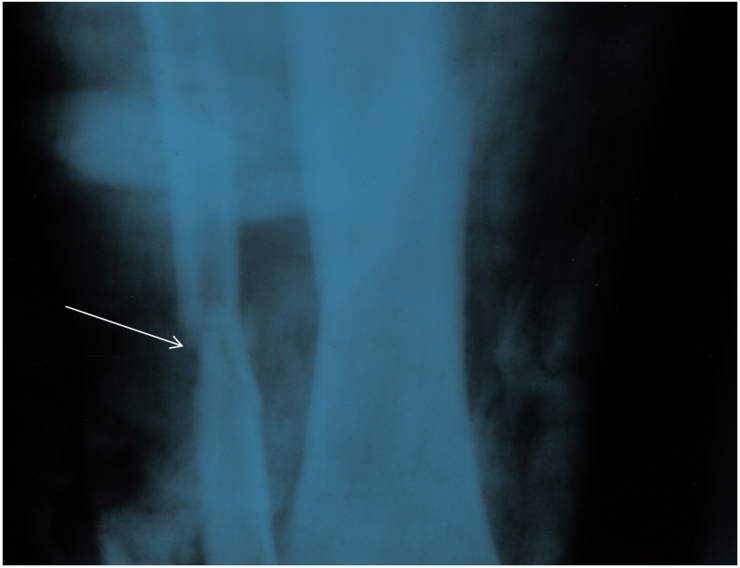


Other physical and clinical examinations revealed that the Superior and inferior right ramous of pubis, proximal of right tibia and shaft of left fibula were fractured (Figure 3,4,5).

Furthermore, an abdominal-pelvic ultrasound (portable) didn’t reveal any abdominal organ laceration or free fluid in abdomen and pelvic areas. Fetus was alive with normal fetal heart rate and amnionic fluid and placenta was in the posterior wall of the uterus without signs of subchorionic hematoma.

**Figure 6: Perineum areas during irrigation-debridement.**
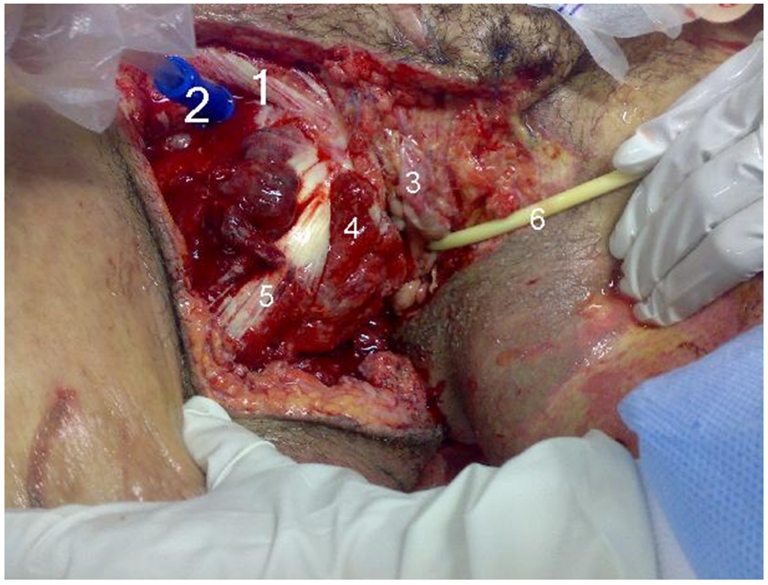
1. Inguinal ligament, 2. The Suction, 3. Labium minus, 4. Bulbospongious muscle, 5. Adductor longus, 6. The Foley Catheter

**The following procedures were included:**

1. Foley catheter was fixed by Urologist (Figure 6). 400 cc clear urine blow out to the urine bag.

2. Induction of anesthesia was started by opioid (fentanyl) and low dose of sodium thiopental. The maintenance of anesthesia was continued by opioid drug.

3. Laparotomy with a midline incision was performed. Abdominal organs exploration were done that revealed an 18 weeks pregnant uterus and no organ damage, but a non-expanding hematoma (sized 10*10 cm) was in the right broad ligament.

4. Diverting Colostomy was done to prevent fecal contamination of the perineum and rectal wound. Secondary infections and necrotizing fasciitis were the main surgical concerns. 

5. Pregnancy was terminated by hysterotomy.

6. Right calcaneal traction and long leg splint were done.

7. The wound of perineum underwent extensive irrigation debridements and it was left open (Figure 4). 

8. After the operation, she was admitted in ICU ward where she started recuperating process.

## Discussion

Traumatic injuries are prevalent in 6% to 7% of pregnant women and are among the most common causes of nonobstetric maternal risk factors for spontaneous abortion, preterm labor, premature delivery, and mortality.^1,2,3,4^Of the causes of trauma, motor vehicle accidents followed by domestic violence and falls are mostly responsible for abdominal injuries in pregnant women in compared to their nonpregnant counterparts. The immediate and long-term management of traumatic injury in pregnant women varies depending on the location and magnitude of the injury. However, regardless of the clinical situation, it is important that clinicians consult with an obstetrician as soon as possible, and perform preoperative assessment that includes airway evaluation as well as rapid and complete resuscitation of the mother. Radiologic tests while properly shielding the pregnant women’s pelvis should remain a viable option as well. Also, when trauma does not result in sudden miscarriage, it is important to inform the patient of the trauma-related potential risks and provide appropriate counseling for the patient.^1,2,3^

If the traumatic injury involves a pregnant women who is in her 1st trimester, it is possible that she is not aware of her pregnancy, therefore, human chorionic gonadotropin test should be performed. This preliminary laboratory test is recommended for all injured women in childbearing age. In cases when traumatic injury occurs during the 2nd or 3rd trimester of pregnancy it is necessary to use ultrasound to verify the age, size, and viability of the fetal. Fetal heart rate should also be regularly monitored to specify how advanced pregnancy is and if the fetus would be viable if delivered.^1,2,3,4^

The advanced trauma life support (ATLS) protocol,^5^depicted in Figure 7 points to the rapid assessment; hemodynamic stabilization; and treatment of maternal injuries as the essential steps for the management of injury and survival of the fetus.^4,5^(Figure 7)

**Figure 7: Simplified assessment and management of a trauma patient. CBC, complete blood count; CT, computed tomography; ECG, electrocardiogram; ED, emergency department; FAST, focused assessment by sonography for trauma; GCS, Glasgow Coma Scale. (Adapted from the Advanced Trauma Life Support curriculum of the American College of Surgeons.):**
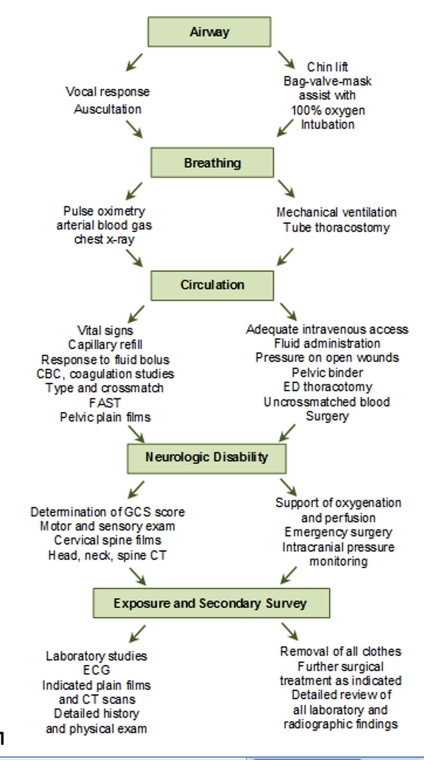


## Specific Aim

The main objective for introducing this case study is to create a platform from which the importance of road traffic related injuries and traumas can be emphasized and discussed within and across various fields of investigation. The long term goal is to entice public campaign around unmet needs for higher road safety measures to reduce primary, secondary, and tertiary risks of injuries and traumas.

We hope that the introduction of this case for a "Ground Round" discussion will stir up a comprehensive discussion regarding the injury and trauma related preventive measures as well as treatment approaches in cases involving pregnant women in car accidents, and will bring about a holistic overview of this issue by the experts in various fields.

## Questions for experts

Questions for experts in potential areas of investigations are outlined below. However, experts from all fields are welcome to submit their feedbacks.

**1. Epidemiology and/or Public Health**

As an epidemiologist and/or a public health specialist/ researcher what would you say are the social, economical, and clinical cost of losing a “Mother”? What are the implications of such fatality (i.e. death of a mother) on the institution of family?  How is Maternal Mortality Rate (MMR) defined and measured in your respective country? Does it include Maternal Mortality cases resulting from Trauma and/or crash injuries? Is it an appropriate decision? Why yes or why not?

**2. Road Safety/ Injury Prevention Experts**

As an expert in improving road safeties, what preventive measures can be implemented to reduce car accident related injuries among pregnant women?

**3. Anesthesiology**

As an anesthesiologist what would have been your  approach for induction and maintenance of anesthesia in this case? Which drugs should have or should have not been prescribed? What would you have done next? What experiences have you personally had regarding similar cases and what were the outcomes?

**4. General Surgery**

As a general surgeon, would you say the above cited approach was appropriate? If you were the surgeon on call what would you have done differently? What would have been your next approach? What experiences have you personally had regarding similar cases and what were the outcomes? 

**5. Orthopedic Surgery**

As an orthopedic surgeon, would you say the above cited approach was appropriate to handle the orthopedic aspect of the case? If you were the orthopedic surgeon on call what would you have done differently? What would have been your next approach? What was your experience in the similar cases? What experiences have you personally had regarding similar cases and what were the outcomes?

**6. Obstetrician and Gynecology**

As an obstetrician/ gynecologist, would you say the above cited approach was appropriate to take care of the Obstetric/Gynecological aspect of the case? If you were the OB/GYN on call what would you have done differently? What would have been your next approach? What experiences have you personally had regarding similar cases and what were the outcomes?

**7. Infectious Disease (ID) and/or Complementary and Alternative Medicine (CAM)**

As an expert in your field (i.e. ID and/or CAM and/or Pathologist and/or Microbiologist etc.) what do you think about the appropriateness of using honey dressing for traumatic open wounds? What experiences have you personally had regarding similar cases and what were the outcomes?

**8. Psychiatry and Psychology**

As an expert in your field of specialty, what are the possible mental health consequences of such a stressful and traumatic experience for the patient? What measures or crisis interventions will you take to prevent possible Depression, PTSD and/or Substance Abuse in this case? What experiences have you personally had regarding similar cases and what were the outcomes?

**9. Other Specialist/Experts**

Others are welcome and invited to send their opinions and comments if additional areas of considerations need to be included in this case report.
